# Reproducibility in Plant Research

**DOI:** 10.1038/s41467-023-38348-1

**Published:** 2023-05-03

## Abstract

Genetic modification is a cornerstone of modern plant biology research and has the potential to transform agriculture. To have the greatest impact, it is essential that the characteristics of new plant genotypes and the methodology used to produce them are accurately reported in the scientific literature. *Nature Communications* is therefore asking for specific methodological details regarding the production of novel plant genotypes to improve transparency and reporting in plant biology.

Since Neolithic times, humans have propagated promising plant materials for food, industry and medicine. The application of scientific methods to this process has uncovered basic tenets of biology and accelerated progress in society. Mendel crossing and cataloguing pea plants laid the groundwork for modern genetics, and breeding efforts during the Green Revolution of the 1950s and 1960s likely saved more than a billion people from starvation.

Fundamental to such efforts is the production and description of novel genotypes by hybridization and mutation. The early 20^th^ century saw efforts to produce new mutants via means such as X-ray radiation and chemical mutagenesis. Targeted approaches such as transgenesis and genome editing followed and have now become mainstays of basic research into understanding gene function and are increasingly used in agricultural biotechnology.

A common complaint from readers and reviewers is that many research papers do not include sufficient details on how such materials are produced. Knowing how a transgenic plant was created and characterized is important when assessing whether a phenotypic change can be attributed to the intended modification. For example, agrobacterium-mediated transformation often introduces multiple transgenes across the genome, which sometimes can disturb the function of endogenous genes where the transgene resides, and so generation of multiple independent lines, backcrossing, or complementation are often needed. In the case of gene editing, a common concern is off-target effects.

Given the diversity of methodologies available and the specific characteristics of each, the details that are reported in research papers and what can be assumed often varies widely. Complicating things further is that terminology differs too—referring to a transformed plant as “T1” may mean different things to different laboratories..

We are therefore introducing a new section on the *Nature Portfolio* Reporting Summary specifically tailored to the characterization of novel plant materials. The Reporting Summary is a checklist that we ask authors to complete once a paper is chosen for formal peer review. The new plant-specific section will ask authors to provide details of the plant materials used in their work. Where new hybrids, mutants or transgenics are reported, we ask that authors provide brief details of the methodology and controls that were used in a standardized form that will be made available to referees and appear online should the paper be published.

The intention is not to be prescriptive - the appropriate methodology and controls will in large part depend on the biological question being examined - but rather to ensure such information is available. We believe this will streamline peer review, benefit authors, reviewers, and readers, and ultimately promote transparency and reproducibility in plant research.

